# Comparison of Nitrate and Perchlorate in Controlling Sulfidogenesis in Heavy Oil-Containing Bioreactors

**DOI:** 10.3389/fmicb.2018.02423

**Published:** 2018-10-09

**Authors:** Gloria Ngozi Okpala, Gerrit Voordouw

**Affiliations:** Department of Biological Sciences, University of Calgary, Calgary, AB, Canada

**Keywords:** sulfate reduction, nitrate, perchlorate, souring, hydrogen sulfide, oil field

## Abstract

Control of microbial reduction of sulfate to sulfide in oil reservoirs (a process referred to as souring) with nitrate has been researched extensively. Nitrate is reduced to nitrite, which is a strong inhibitor of sulfate-reducing bacteria (SRB). Perchlorate has been proposed as an alternative souring control agent. It is reduced to chlorate (ClO_3_^-^) and chlorite (ClO_2_^-^), which is dismutated to chloride and O_2_. These can react with sulfide to form sulfur. Chlorite is also highly biocidal. Here we compared the effectiveness of perchlorate and nitrate in inhibiting SRB activity in medium containing heavy oil from the Medicine Hat Glauconitic C (MHGC) field, which has a low reservoir temperature and is injected with nitrate to control souring. Using acetate, propionate and butyrate as electron donors, perchlorate-reducing bacteria (PRB) were obtained in enrichment culture and perchlorate-reducing *Magnetospirillum* spp. were isolated from MHGC produced waters. In batch experiments with MHGC oil as the electron donor, nitrate was reduced to nitrite and inhibited sulfate reduction. However, perchlorate was not reduced and did not inhibit sulfate reduction in these incubations. Bioreactor experiments were conducted with sand-packed glass columns, containing MHGC oil and inoculated with an oil-grown mesophilic SRB enrichment. Once active souring (reduction of 2 mM sulfate to sulfide) was observed, these were treated with nitrate and/or perchlorate. As in the batch experiments, 4 mM nitrate completely inhibited sulfide production, while partial inhibition occurred with 1 and 2 mM nitrate, but injection of 4 mM perchlorate did not inhibit sulfate reduction and perchlorate was not reduced. The enriched and isolated PRB were unable to use heavy oil components, like alkylbenzenes, which were readily used by nitrate-reducing bacteria. Hence perchlorate, injected into a low temperature heavy oil reservoir like the MHGC, may not be reduced to toxic intermediates making nitrate a preferable souring control agent.

## Introduction

Waterflooded low temperature reservoirs have a constant temperature from injector to producing wells, allowing growth of different mesophilic bacteria or archaea depending on the electron acceptors present. The presence of sulfate in injection water stimulates the growth of sulfate-reducing bacteria (SRB), which reduce sulfate to sulfide (H_2_S or HS^-^) resulting in reservoir souring. H_2_S is a toxic and corrosive gas, which constitutes a health hazard to oilfield operators. Corrosion of carbon steel infrastructure, contamination of produced oil and reservoir plugging due to precipitation of metal sulfides are other souring-related problems (Vance and Thrasher, 2005). A commonly used control measure for reservoir souring is the injection of nitrate. This is reduced by nitrate-reducing bacteria (NRB) to nitrite and then mostly to N_2_. Nitrite, a strong metabolic inhibitor of SRB, binds to dissimilatory sulfite reductase which catalyzes the reduction of sulfite to sulfide as the last step in the sulfate reduction pathway. However, many NRB reduce nitrite further to dinitrogen (N_2_) removing the inhibition. Continuous injection of sulfate-containing, nitrate-amended water into a low temperature reservoir leads, therefore, to microbial zonation where NRB activity is limited to the near-injection wellbore region (NIWR) with SRB activity occurring deeper into the reservoir (Voordouw et al., 2009; Callbeck et al., 2011). Deeper zones of sulfate reduction may be controlled by the batch-wise injection of high concentrations of nitrate to push nitrate deeper into the reservoir (Voordouw et al., 2009; Callbeck et al., 2011) or by the combined injection of nitrate and biocides (Xue and Voordouw, 2015; Fida et al., 2018).

Perchlorate has been proposed as an alternative to nitrate in the control of reservoir souring (Coates, 2014; Liebensteiner et al., 2014). Perchlorate was first reported by Postgate (1952) to directly inhibit sulfate reduction in *Desulfovibrio desulfuricans*, when added at 10 times the concentration of sulfate in the growth medium. Since then, anaerobic reduction of perchlorate and chlorate has been studied extensively and the mechanism by which perchlorate inhibits sulfate reduction has been elucidated. Perchlorate- and/or chlorate-reducing bacteria (PRB) inhibit SRB via (i) biocompetitive exclusion (Engelbrektson et al., 2014; Liebensteiner et al., 2014), (ii) oxidation of sulfide to sulfur (Coates and Achenbach, 2004; Gregoire et al., 2014; Mehta-Kolte et al., 2017), and/or (iii) direct inhibition of the dissimilatory sulfate respiration pathway (Carlson et al., 2015). In (ii) sulfide is oxidized either biotically through enzyme-catalyzed reactions starting with perchlorate reductase (PcrAB) or through the abiotic reaction of sulfide with chlorite or oxygen formed as intermediate reaction products in microbial perchlorate reduction (Mehta-Kolte et al., 2017). Inhibition of sulfide production by perchlorate in a bioreactor column injected with sulfate and yeast extract has been reported by Engelbrektson et al. (2014). No sulfide was detected in the column effluent and perchlorate injection stimulated the emergence of a diverse microbial community (Engelbrektson et al., 2014).

Studies on the control of sulfide production with nitrate in heavy oil-containing bioreactor columns as well as in the field have shown that alkylbenzenes (toluene, ethylbenzene, and *m*- and *p*-xylene) were utilized as electron donors for nitrate reduction (Lambo et al., 2008; Agrawal et al., 2012; Suri et al., 2017), whereas alkylbenzenes and alkanes were used as electron donors for sulfate reduction (Agrawal et al., 2012). Acetate accumulated transiently as an intermediate in the metabolism of these oil components under sulfate-reducing, but not under nitrate-reducing conditions (Callbeck et al., 2013).

It is presently unknown whether perchlorate can prevent souring in oil-containing bioreactors and, by extension, in oil reservoirs. *Dechloromonas* strain RCB uses benzene as electron donor for perchlorate reduction (Coates et al., 2001; Chakraborty et al., 2005), but oxidized alkylbenzenes only under nitrate-reducing conditions. *Pseudomonas chloritidismutans* AW-1(T) couples the reduction of chlorate (not perchlorate) to the oxidative metabolism of n-alkanes (Mehboob et al., 2009, 2016). This organism activates hydrocarbon with a mono-oxygenase using oxygen generated by the dismutation of chlorite by the enzyme chlorite dismutase (Wolterink et al., 2002; Mehboob et al., 2009, 2016). Hence, the potential of PRB for controlling souring with perchlorate in bioreactors with oil as the sole electron donor is promising. The objective of the current work was, therefore, to directly compare control of sulfide production in low-temperature, oil-containing bioreactors with nitrate and perchlorate.

## Materials and Methods

### Source of Field Samples

Produced water samples were obtained from production well 18PW of the Medicine Hat Glauconitic C (MHGC) field near Medicine Hat, Alberta. This is a shallow reservoir (850 m below the surface; reservoir temperature 30°C) from which heavy oil with an American Petroleum Institute (API) gravity of 16° is produced by re-injection of produced water. The water samples were collected in sterile 1 L Nalgene bottles filled to the brim to exclude air during transport. Following arrival at the University of Calgary within half a day from collection, samples were transferred to a Coy anaerobic hood with an atmosphere of 90% N_2_ and 10% CO_2_ (N_2_–CO_2_).

### Batch Culture Enrichments

Anaerobic Coleville synthetic brine medium K (CSBK; Fida et al., 2016), pH 7.5, was dispensed in 20 mL aliquots in 60 mL serum bottles with a head space of N_2_–CO_2_, sealed with butyl rubber stoppers and aluminum rings. This medium was amended with electron acceptors (e.g., sulfate, perchlorate, or nitrate) and electron donors like 3 mM volatile fatty acids (VFA, 3 mM each of acetate, propionate and butyrate), 3 mM toluene, 2 mM ethylbenzene, or 1 mL of MHGC oil, as indicated. Batch cultures were inoculated with 5% (v/v) of 18PW unless stated otherwise and were incubated at 30°C.

Serum bottles used to determine the time course of growth with VFA and perchlorate, VFA and nitrate, or VFA and nitrate and perchlorate were inoculated with a PRB enrichment. Samples (1 mL) were taken periodically using N_2_–CO_2_ flushed syringes to determine growth (OD_600_), the concentrations of nitrite (NO_2_^-^), nitrate (NO_3_^-^), and perchlorate (ClO_4_^-^) and to isolate DNA for community analysis.

### Inhibition of Oil-Dependent Sulfate Reduction With Perchlorate and/or Nitrate in Batch Cultures

Aliquots (20 mL) of anaerobic CSBK medium in 60 mL serum bottles were amended with 1 mL of MHGC oil (O) and either 5 mM sulfate (S), 5 mM sulfate and 10 mM nitrate (SN), 5 mM sulfate and 10 mM perchlorate (SP), 5 mM sulfate and 10 mM nitrate and 10 mM perchlorate (SNP), or 10 mM perchlorate (P). Bottles were inoculated with 1 mL of 18PW, concentrated 20-fold by centrifugation (Okpala et al., 2017). Sterile controls, containing 20 mL of CSBK medium, 1 mL of MHGC oil and electron acceptors, but no inoculum, were also prepared. Concentrations of sulfate, nitrate, nitrite, perchlorate, and aqueous sulfide were determined as a function of time in duplicate incubations. The concentrations of residual alkylbenzenes and alkanes present in dichloromethane (DCM) extracts of the residual oil were determined using gas chromatography-mass spectrometry (GC-MS).

### Control of Souring With Nitrate and/or Perchlorate in Oil-Containing Bioreactors

Glass syringe bioreactors (50 mL) were assembled essentially as described by Callbeck et al. (2011). Bioreactors were sealed at the bottom with a layer of glass wool and polymeric mesh and were tightly packed with silica sand (Sigma-Aldrich, 50–70 mesh). The upper ends were sealed with a layer of polymeric mesh and a rubber stopper perforated with a 1 mL syringe through which effluent flowed into an effluent collector. Sterile anaerobic CSBK from a medium reservoir was injected through the inlet bottom end of the bioreactor using a Minipuls-3 multi-channel peristaltic pump (Gilson Inc.). The medium reservoir was maintained anaerobic through injection of N_2_–CO_2_ in the headspace. Once the columns were saturated with medium, their wet weight was determined to calculate their pore volumes (PV, wet weight minus dry weight), which was approximately 25 mL for each column. The columns were then flooded first with 1 PV of MHGC oil and then with multiple PV of anoxic CSBK medium until approximately 0.5 PV of oil was produced, as determined spectrophotometrically (Gassara et al., 2015). At that point little further oil production was observed and approximately 0.5 PV of oil remained in the columns. The columns were then inoculated with SRB culture grown with MHGC oil and sulfate. The three-way valves at the inlet and outlet ends were closed and the columns were incubated for 20 days. Following incubation, injection of anoxic medium containing 2 mM sulfate was resumed at a flow rate of 0.5 PV/day. Concentrations of sulfate, sulfide and other anions tested were monitored for every 1 PV of effluent.

### Reduction of Perchlorate or Nitrate With Alkylbenzene Electron Donors

Perchlorate and nitrate reduction with toluene or ethylbenzene were compared in serum bottles with 20 mL of CBSK medium, containing 10 mM perchlorate or 10 mM nitrate and 3 mM of aqueous toluene or 2 mM of aqueous ethylbenzene. Alternatively, 1 mL of 60 mM toluene or 40 mM ethylbenzene in the inert carrier 2,2,4,4,6,8,8-heptamethylnonane (HMN) was added. The inoculum used was either 1 mL of 20-fold concentrated 18PW or 2 mL of a chemostat culture derived from 18PW, which was growing on toluene and nitrate or on ethylbenzene and nitrate.

### Isolation and Identification of PRB

Perchlorate-reducing bacteria enrichments obtained with VFA and perchlorate at 30°C were diluted 10-fold and plated on CSBK medium with 3 mM VFA and 10 mM perchlorate, solidified with 15 g/L of agar. The plates were incubated at 30°C in anaerobic jars flushed with N_2_–CO_2_. Individual colonies were picked and transferred to CSBK medium with 3 mM VFA and 10 mM perchlorate. The isolates were phylogenetically identified by Sanger sequencing of 1500-bp 16S rRNA gene amplicons obtained with universal primers 27F and 1525R (Frank et al., 2008) at the Core DNA Services Laboratory of the University of Calgary. The 16S rRNA gene sequences of the isolates and of reference sequences, retrieved from GenBank, were aligned with Clustal W (Thompson et al., 1994). A phylogenetic tree was constructed using MEGA version 6 (Tamura et al., 2013). The evolutionary history was inferred using the Neighbor-Joining algorithm (Saitou and Nei, 1987). The evolutionary distances were computed using the Maximum Composite Likelihood method (Tamura et al., 2004) in units of the number of base substitutions per site. Confidence estimates of branch clusters were obtained from bootstrap tests of 1000 replicates (Felsenstein, 1985).

### Microbial Community Analysis by Illumina Sequencing

DNA was isolated from enrichments using the Fast DNA Spin Kit for Soil and the FastPrep Instrument (MP Biomedicals, Santa Ana, CA, United States) as per the manufacturer’s instructions. The extracted DNAs were quantified, with the Quant-iT^TM^ dsDNA HS assay kit using a Qubit fluorimeter (Invitrogen) and were subjected to a two-step PCR amplification of 16S rRNA. PCR reactions were carried out in duplicate with each replicate containing a 20 μl reaction volume. The amplified PCR products were pooled and cleaned using the QIAquick PCR purification kit (Qiagen) and analyzed on a 1.5% agarose gel. In the first step non-barcoded primers 926Fi5 and 1392Ri7 were used, while the barcoded primers P5-S50X-OHAF and P7-N7XX-OHAF were used in the second step. PCR conditions were 95°C for 5 min, followed by 25 cycles of 95°C for 45 s, 55°C for 2 min, 72°C for 4 min, followed by a final incubation at 72°C for 10 min in the first step and 95°C for 3 min, followed by 10 cycles of 95°C for 45 s, 55°C for 2 min, 72°C for 4 min, followed by a final incubation at 72°C for 10 min in the second step. The final concentrated PCR products were diluted with the Qiagen elution buffer to 4 ng/μl. The 16S amplicons were sequenced using the 300PE (paired-end) MiSeq protocol of the Illumina Miseq system at the Energy Bioengineering and Geomicrobiology Group (EBG) of the University of Calgary.

Analyses of sequencing data involved merging the 300PE reads from both ends with a minimum overlap of 50 bp and a minimum length of 450 bp as cut-offs using the PEAR 0.9.6 software. The merged reads were processed with MetaAmp (Dong et al., 2017b). The sequencing data retrieved were clustered into operational taxonomic units (OTUs) at a taxonomic distance of 3%. Rarefaction curves and alpha diversity indices were calculated including Chao1 (Chao, 1984) and Shannon’s H-index (Shannon, 1948). Relational trees showing the beta diversity of the amplicon sequences were visualized using MEGA 6 (Tamura et al., 2013). All sequences have been submitted to NCBI Sequence Read Archive (SRA) under Bioproject accession number PRJNA181037 with biosample numbers SAMN09519490 to SAMN09519497 and SAMN09521198 to SAMN09521209.

### Analytical Techniques

Samples for anion assays were prepared by centrifugation at 14,000 ×*g* for 5 min, after which 100 μl of supernatant was added to 400 μl of the prepared buffer solution in a vial. Concentrations of NO_3_^-^, NO_2_^-^, and ClO_4_^-^ in the samples were analyzed by high-pressure liquid chromatography (HPLC) using a Waters 600E instrument (Waters Corp, Milford, MA, United States), which was fitted with an IC-PAK^TM^ anion HC column (150 mm × 4.6 mm, Waters) and a Waters 2489 UV/Visible detector, set at 220 nm. Nitrate and nitrite were eluted with a sodium borate-gluconate buffer containing 12% (v/v) acetonitrile and 2% (v/v) butanol at a flow rate of 2 mL/min. Perchlorate was eluted using an alkaline buffer mixture of 25 mM NH_4_HCO_3_ in 50% (v/v) acetonitrile, pH 10 (adjusted with NH_4_OH) at a flowrate of 1.5 mL/min. Perchlorate was detected with a Waters 432 conductivity detector using the same column as above. The concentration of VFA components was analyzed with a Prevail organic acid (OA) 5u column (250 mm × 4.6 mm, Alltech, Guelph, ON, Canada) fitted with a Waters 2487 UV detector at 210 nm.

### Gas Chromatography-Mass Spectrometry (GC-MS) Analysis of Oil Components

Extraction of oil components with dichloromethane (DCM) was done as described by Agrawal et al. (2012). Prior to DCM extraction of the oil, 50 μL of squalene and mesitylene were added to the 1 mL oil layer of each incubation as internal standards for quantifying the depletion of *n*-alkanes and alkylbenzenes, respectively. Following this, 9 mL of DCM was added to extract the oil components and one microliter (μL) of the oil-DCM layer was injected by an autoinjector (7683B series, Agilent Technologies) into a GC (7890N series, Agilent Technologies), connected to an MS (5975C inert XL MSD series, Agilent). The GC was equipped with an HP-1 fused silica capillary column (length 50 m, inner diameter 0.32 mm, film thickness 0.52 μm; J&W Scientific) with helium as carrier gas. The amounts of oil components utilized for the reduction of the electron acceptors were determined as the decrease in the ratio of the peak area for a given component to that of the internal standard.

## Results

### Activity of PRB in the 18PW Field Sample

**FIGURE 1:**
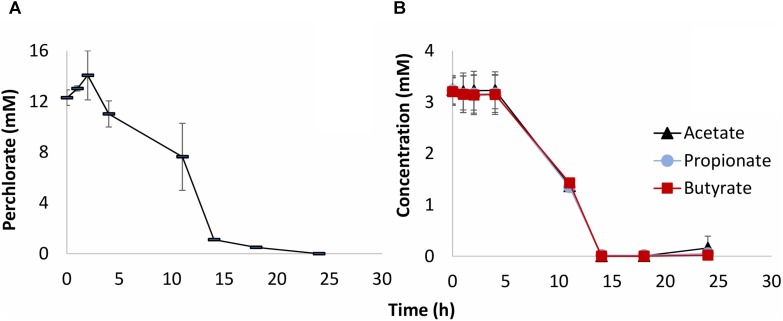
Activity of PRB in CSBK medium with 10 mM perchlorate and 3 mM VFA, inoculated with 5% (v/v) of produced water sample 18PW and incubated at 30°C. The concentrations of perchlorate **(A)** of VFA **(B)** are shown as a function of time (h). The data presented are averages of duplicate incubations.

The results of inoculation of produced water 18PW from the MHGC field into CSBK medium with VFA and perchlorate and incubation at 30°C are shown in **Figure 1**. Perchlorate was reduced after a 4-day lag phase with complete reduction after 24 days of incubation (**Figure 1A**). All three VFA (acetate, propionate, and butyrate) were used for perchlorate reduction (**Figure 1B**), which indicates their suitability as electron donors for reduction of perchlorate in the MHGC field.

### Growth and Community Composition of Enrichments With Nitrate and/or Perchlorate

**FIGURE 2:**
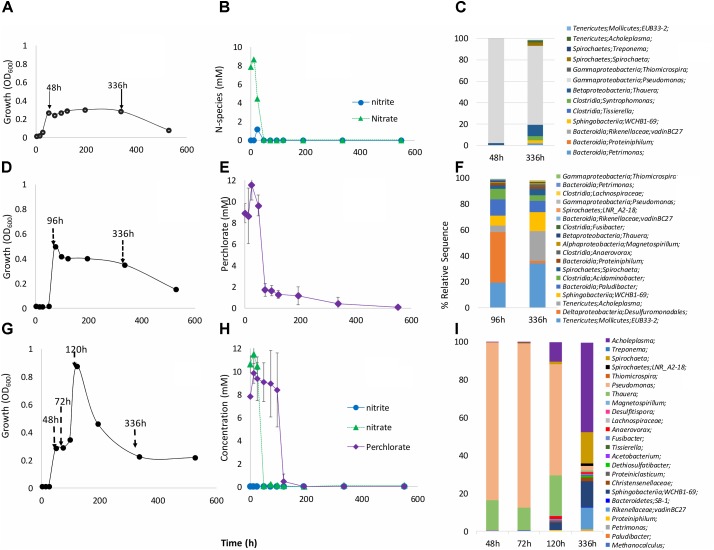
Time course of nitrate **(A–C)**, perchlorate **(D–F)** or nitrate and perchlorate **(G–I)** reduction in 18PW communities incubated in CSBK medium containing 10 mM each of nitrate and/or perchlorate and VFA. **(A,D,G)** Growth OD at 600 nm; **(B,E,H)** concentrations of electron acceptors; **(C,F,I)** microbial community composition at *T* = 48, 72, 96, 120, and 336 h, as indicated (**Supplementary Table S1**).

The primary enrichment with VFA and perchlorate (**Figure 1**) was used to inoculate secondary enrichments in media with VFA and nitrate, VFA and perchlorate, or VFA and nitrate and perchlorate (**Figure 2**). In cultures with VFA and nitrate growth was indicated as an increase in OD_600_ to 0.27 at 42 h after which the OD_600_ was relatively constant up to 336 h (**Figure 2A**). Nitrate was completely reduced at 48 h with transient formation of 1.2 mM nitrite (**Figure 2B**). Microbial community compositions at 48 h indicated *Pseudomonas* (97%) and *Thauera* (2%), which are genera of known heterotrophic NRB (hNRB). At 336 h the fraction of *Pseudomonas* had decreased to 74%, whereas that of *Thauera* had increased to 10.5% (**Figure 2C** and **Supplementary Table S1**). Increases were also seen for the genera *Petrimonas* (0.01–1.74%) and *Acholeplasma* (0.15–1.7%).

In enrichments with perchlorate a peak OD_600_ of 0.50 was observed at 96 h (**Figure 2D**). Most perchlorate in the medium was reduced during this period with a slower reduction observed from 96 to 336 h (**Figure 2E**). The microbial community in the perchlorate-reducing enrichment was more diverse, than that in the nitrate-reducing enrichment (**Supplementary Table S1**: Shannon indices of 1.93 and 2.06 and of 0.18 and 1.13, respectively). The dominant community members at 96 h (**Figure 2F** and **Supplementary Table S1**) were *Desulfuromonadales* (39.2%), *Mollicutes* EUB33-2 (19.3%), *Acholeplasma* (5.0%), *Acidaminobacter* (7.9%), *Sphingobacteria* WCHB1-69 (7.4%), *Paludibacter* (12.7%), and *Magnetospirillum* (0.8%). At 336-h increased fractions were observed for the *Mollicutes* (34%), *Sphingobacteria* (14.3%), and *Acholeplasma* (23.1%).

In medium containing both nitrate and perchlorate, biphasic growth was observed as perchlorate reduction was delayed until nitrate reduction was complete (**Figures 2G,H**). Initial nitrate-reducing growth occurred from 0 to 48 h, with OD_600_ = 0.27 at 48 h. This was followed by perchlorate-reducing growth from 72 to 120 h with OD_600_ = 0.88 at 120 h (**Figure 2G**). These increases in OD_600_ (0.27 and 0.51 for nitrate and perchlorate) were very similar to those observed in cultures with only nitrate or perchlorate. Examination of early microbial community compositions showed dominance of *Pseudomonas* (83% at 48 h and 86% at 72 h) and *Thauera* (16% at 48 h and 11.8% at 72 h). From 120 to 336 h the taxa *Acholeplasma* (10.4 to 47.2%), *Sphingobacteria* (4 to 13.8%), *Fusibacter* (0.85 to 0.46%), and *Rikenellaceae* (0.12 to 11.6%) were observed (**Figure 2I** and **Supplementary Table S1**).

### Effects of Perchlorate and Nitrate on Sulfide Production in Batch Cultures With Oil

Batch incubations were started to determine the effect of nitrate, perchlorate, or nitrate and perchlorate on sulfate reduction in CSBK medium containing 1 mL of MHGC oil and inoculated with concentrated 18PW. Changes in the composition of the microbial community of selected oil components were also monitored.

**FIGURE 3:**
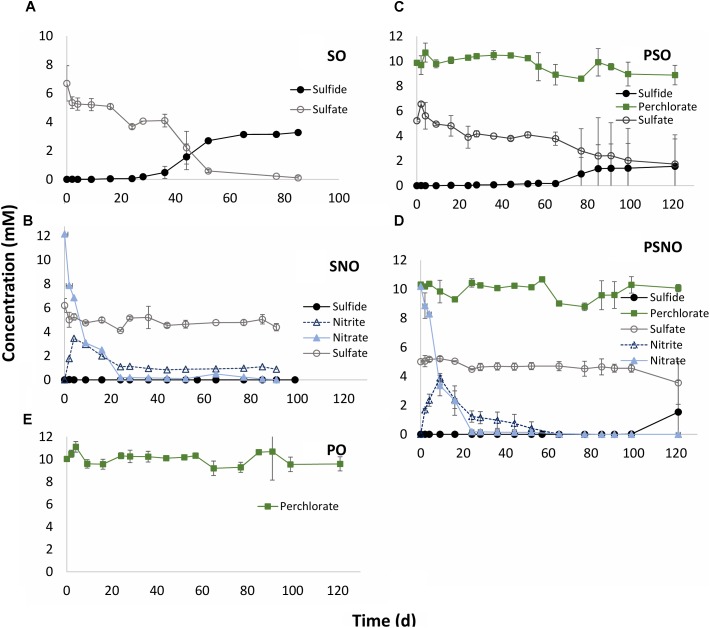
Inhibition of sulfide production by oil grown SRB with nitrate, perchlorate or nitrate, and perchlorate. CSBK medium containing 1 mL MHGC oil (represented as O) as electron donor was inoculated with a 20-fold concentrated 18PW sample. Medium was amended with 5 mM sulfate – SO **(A)**; 5 mM sulfate and 10 mM nitrate – SNO **(B)**; 5 mM sulfate and 10 mM perchlorate – PSO **(C)**; 5 mM sulfate and 10 mM each of perchlorate and nitrate – PSNO **(D)**; and 10 mM of perchlorate only – PO **(E)**. Data presented are means of duplicate incubations.

Sulfate reduction with oil as electron donor proceeded slowly. Following a lag phase of 24 days reduction of 5 mM sulfate to 3.3 mM aqueous sulfide was observed after 77 days of incubation (**Figure 3A**: SO). In the presence of 10 mM nitrate, sulfate reduction was completely inhibited. Nitrate was completely reduced after 24 days, with peak nitrite (3.5 mM) occurring at day 9. The nitrite concentration was further decreased to 1 mM at day 24 and this concentration persisted preventing the start of sulfate reduction (**Figure 3B**; SNO). In the presence of 10 mM perchlorate a longer lag phase of 65 days was observed before the start of sulfate reduction, indicating some inhibition of sulfate reduction. However, sulfate reduction was not prevented whereas perchlorate was not reduced (**Figure 3C**: PSO). No perchlorate reduction was observed in incubations in which only perchlorate and oil were present (**Figure 2E**: PO). In incubations with all three electron acceptors (nitrate, sulfate, and perchlorate) nitrate was reduced first with transient formation of 4 mM nitrite. Nitrate and nitrite were completely reduced after 34 and 65 days, respectively. Perchlorate reduction was not observed and was unable to prevent reduction of sulfate to sulfide, which started after 100 days (**Figure 3D**: PSNO).

Analysis of the alkylbenzenes toluene, ethylbenzene, m,p-xylene and o-xylene remaining in the oil after 123 days of incubation showed that these were mostly used in incubations with sulfate and that these were completely used in incubations with sulfate and nitrate with the exception of o-xylene (**Supplementary Figure S1**: SO and SNO). These alkylbenzenes were not used in incubations with perchlorate only (**Supplementary Figure S1**: PO). Their use in incubations with sulfate and perchlorate and with sulfate, nitrate, and perchlorate (**Supplementary Figure S1**: PSO and PSNO) was, therefore, for reduction of sulfate and of sulfate and nitrate, respectively, not for reduction of perchlorate.

### Microbial Community Compositions in Batch Cultures With Oil

**FIGURE 4:**
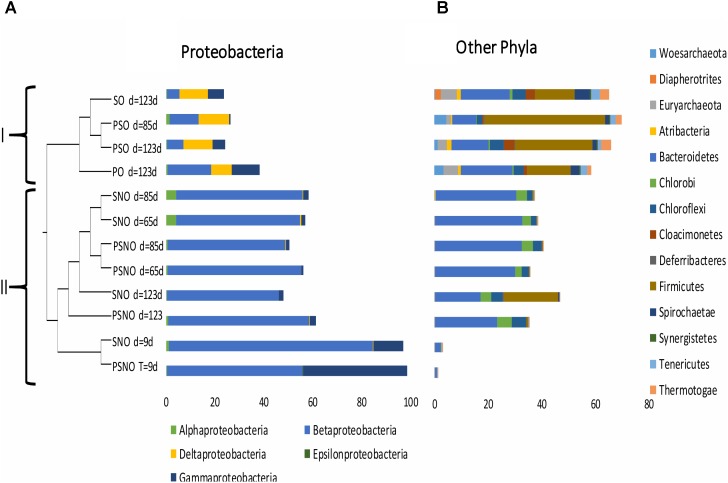
Relational tree for 16S rRNA gene libraries from incubations of CSBK medium, inoculated with 18PW and amended with 1 mL MHGC oil and sulfate (SO); sulfate and nitrate (SNO); sulfate and perchlorate (PSO); sulfate, nitrate, and perchlorate (PSNO); or perchlorate only (PO). Community DNA was obtained from cells harvested at day 9, 65, 85, or 123 as indicated. The fractions of reads in classes of *Proteobacteria* **(A)** and in other phyla **(B)** are shown.

Illumina sequencing of 16S rRNA amplicons and analysis of the sequences obtained indicated that communities in incubations with nitrate were less diverse than those in incubations without nitrate (**Supplementary Table S2**: Shannon indices of 0.76–2.04 and 2.82–3.93, respectively). Likewise, comparison of microbial communities derived from these sequenced amplicons in a dendrogram indicated that communities in incubations without nitrate clustered separately from communities in incubations with nitrate (**Figure 4**: clusters I and II, respectively). In cluster I communities from incubations with sulfate (SO) and sulfate and perchlorate (PSO) were distinct from incubations with perchlorate only (PO), whereas in cluster II communities in short term incubations (9 days) were distinct from those in longer term incubations (65–123 days). At the class level, cluster I had higher fractions of *Deltaproteobacteria*, whereas cluster II had higher fractions of *Beta-* and *Gammaproteobacteria* (**Figure 4A**). Communities in cluster I also had higher fractions of the phyla *Atribacteria*, *Euryarchaeota*, *Firmicutes*, and *Spirochaetae* (**Figure 4B**). Unique to cluster I was the candidate phylum *Cloacimonetes*, which is mostly found in anaerobic environments, often in close association with *Firmicutes* (Stolze et al., 2016). *Cloacimonetes* have been predicted to be syntrophic propionate fermenters (Hamilton et al., 2016) producing CO_2_ and H_2_, which is used by hydrogenotrophic methanogens or SRB (Stolze et al., 2016).

At the genus level all nitrate-reducing communities in cluster II had high fractions of *Thauera* (**Supplementary Table S2**, entry #1: 44–82%), whereas high fractions of *Pseudomonas* were observed in early communities (**Supplementary Table S2**, entry #4: 12–43%). All communities in cluster I had high fractions of *Smithella* (**Supplementary Table S2**, entry #9: 2.8–6.5%) and *Desulfomicrobium* (**Supplementary Table S2**, entry #18: 0.9–2.4%). However, the putative SRB *Desulfarculus*, *Desulfobacteraceae*, and *Desulfocurvus* were mostly present in the SO and PSO and not in the PO incubations (**Supplementary Table S2**, entries #16, 20, and 22). *Pelotomaculum* of the phylum *Firmicutes*, identified in the SO and PSO incubations (**Supplementary Table S2**, entry #3: 11–40%), was also lacking in the PO incubations. Dong et al. (2017a) showed that a *Pelotomaculum* sp., which coupled the reduction of sulfate to the anaerobic oxidation of benzene, possessed genes for dissimilatory sulfate reduction as found in other Gram-positive SRB. In contrast, the microbial community in the PO incubations did not have taxa that were absent in the SO and PSO incubations, which is consistent with the fact that the communities in the PO incubations did not catalyze perchlorate reduction.

### Souring Control in Oil-Containing Bioreactors

Eight sand packed columns containing 0.5 PV of residual MHGC oil, were set up to evaluate the control of sulfide production by SRB using nitrate, perchlorate or a combination of these. Duplicate 50 mL glass columns were used for each treatment condition, and two columns remained untreated. To ensure uniformity, all the columns were inoculated with the same SRB culture enriched with MHGC oil and 2 mM sulfate. Following a 20-day incubation period, these columns were flooded continuously with anaerobic CSBK medium, containing 2 mM sulfate and monitored until approximately 2 mM sulfide was produced. Once souring had been established, treatment with either nitrate or perchlorate was started.

**FIGURE 5:**
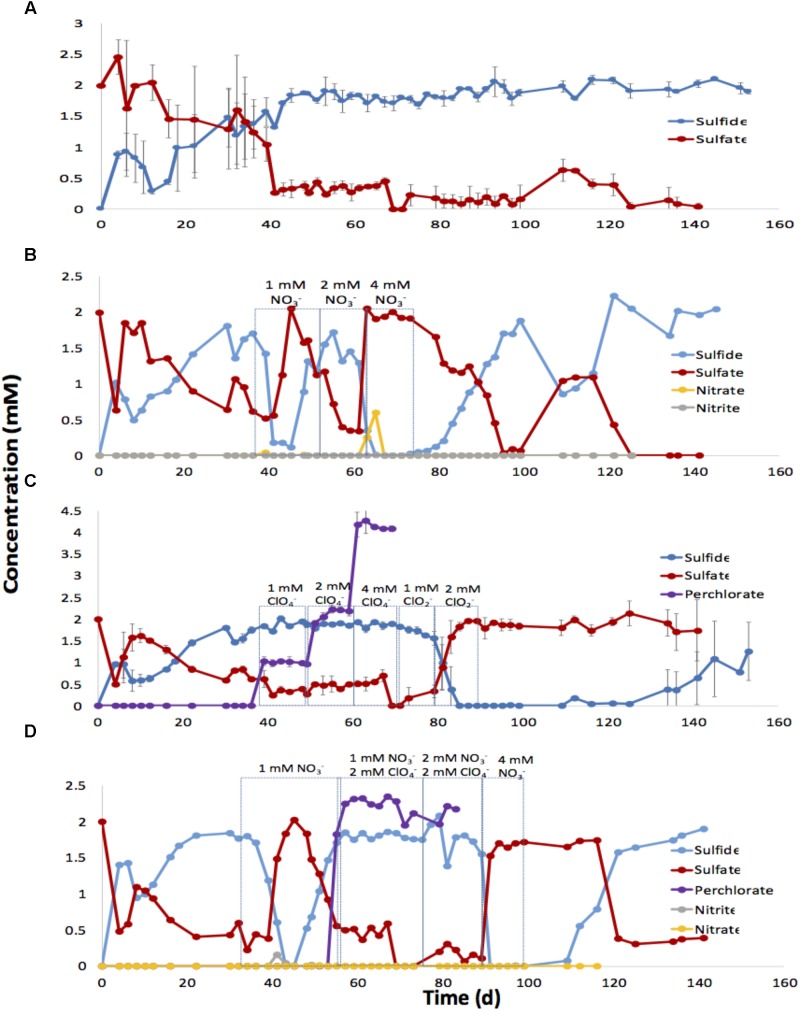
Effect of nitrate, perchlorate or chlorite, or nitrate and perchlorate on sulfide production in low temperature, oil-containing bioreactors. Bioreactors were inoculated with SRB enrichments of 18PW grown in CBSK medium with sulfate and MHGC oil. Bioreactors were continuously injected with CBSK medium containing 2 mM sulfate at a flowrate of 0.5 PV/day. The effluent concentrations of sulfate, sulfide, nitrate, nitrite, and perchlorate are shown for untreated columns **(A)**, columns injected with nitrate **(B)**, columns injected with perchlorate or chlorite **(C)**, and columns injected with nitrate and perchlorate **(D)** as a function of time.

In the untreated columns sulfide was observed on day 4 (0.88 ± 0.06 mM), reaching 1.8 ± 0.11 mM on day 45. Production of up to 2 mM sulfide was then maintained for the duration of the experiment up to day 150 (**Figure 5A**). Injection of soured duplicate columns with 2 mM sulfate and 1 mM nitrate decreased sulfide to 0.1 mM for the first 2 PV after which sulfide production recovered. Increasing the nitrate concentration to 2 mM also caused only transient partial inhibition of sulfide production. However, when the injected nitrate concentration was increased to 4 mM, sulfide production was completely inhibited and the measured effluent sulfate concentrations reached 2 mM. Nitrate (0.6 mM) was detected in the first PV of bioreactor effluent, whereas nitrite was not detected (**Figure 5B**). When nitrate injection was stopped, the effluent sulfide concentration recovered to 2 mM by day 100.

Injection of perchlorate at increasing concentrations of 1, 2, or 4 mM did not control the activity of SRB in the bioreactors as shown in **Figure 5C**. As in batch cultures incubation with oil, perchlorate was not reduced in the bioreactors and the injected concentrations were fully recovered in the effluent. Since perchlorate showed no inhibition of SRB activity, chlorite was injected instead (**Figure 5C**). Injection of 1 mM chlorite decreased the effluent sulfide concentration from 1.9 to 1.6 mM, whereas injection of 2 mM chlorite completely inhibited sulfide production by SRB in the bioreactors. Recovery of SRB activity, following the cessation of the chlorite injection, required injection of 28 PV of the CSBK medium with 2 mM sulfate to reach 1 mM sulfide (**Figure 5C**).

Combined injection of nitrate and perchlorate is shown in **Figure 5D**. Injection of 1 mM nitrate only caused transient inhibition of sulfide production. Sulfide concentrations decreased from 1.8 to 0 mM following injection of the first 5 PV and then recovered to 1.8 mM. Nitrate was reduced in the bioreactors and only nitrite was detected transiently in the effluent. Combining 2 mM perchlorate with 1 or 2 mM nitrate did not increase inhibition of sulfate reduction, as effluent sulfide remained at 1.8 mM. Switching the injection medium to CSBK with 4 mM nitrate and 2 mM sulfate gave immediate inhibition of sulfide production and increased the effluent sulfate concentration to 1.7 mM. Sulfate reduction resumed 22 days after nitrate injection was stopped (**Figure 5D**).

### Reduction of Nitrite and Chlorite in the Presence of Oilfield Microbes

**FIGURE 6:**
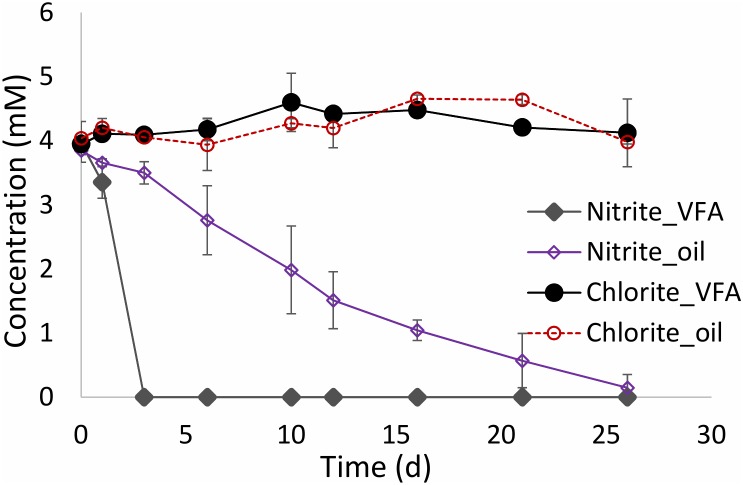
Comparison of reduction of nitrite and chlorite in medium containing VFA or MHGC oil and inoculated with 10% (v/v) 18PW.

Nitrite and chlorite are potentially inhibitory products of nitrate and perchlorate reduction. In order to compare their fate directly, batch cultures containing 50 mL of CSBK medium, 1 mL MHGC oil or 3 mM VFA and 4 mM nitrite or 4 mM chlorite, were inoculated with 10% (v/v) 18PW and incubated at 30°C for 30 days. The results indicated that oil field hNRB rapidly reduced nitrite with VFA, while nitrite reduction with oil progressed more slowly over a 26-day period (**Figure 6**). Chlorite on the other hand was not reduced and remained at a constant concentration in the presence of VFA or oil (**Figure 6**). Chlorite can be disproportionated to chloride and oxygen through a non-energy yielding reaction, catalyzed by chlorite dismutase (Rikken et al., 1996; van Ginkel et al., 1996; Bender et al., 2005; Bardiya and Bae, 2011; Schaffner et al., 2015). However, this did not occur in the incubations in **Figure 6**. Chlorite is a strong oxidant, which reacts non-specifically with biomolecules resulting in cell damage and death (McDonnell and Russell, 1999). The results suggest that 4 mM chlorite killed the microbes present in the incubations; chlorite did not react abiotically with either oil or VFA. Hence, nitrite and chlorite behaved very differently with nitrite having less acute toxicity than chlorite.

### Search for Alkylbenzene-Oxidizing PRB

Although perchlorate concentrations remained unchanged in batch culture and bioreactor experiments with oil, some further attempts were made to enrich microbes using the alkylbenzenes toluene or ethylbenzene as electron donor for perchlorate reduction. Alkylbenzenes were added directly to 50 mL aqueous CSBK medium or to 1 mL of HMN overlaying this medium to minimize toxicity. A 20-fold concentrated 18PW sample (1 mL) or a nitrate-reducing chemostat culture (5 mL), continuously fed with either toluene or ethylbenzene (Suri, 2017), were used as inocula. However, these were unable to use toluene or ethylbenzene for reduction of 10 mM perchlorate within 30 days of incubation. When incubations inoculated with chemostat culture were amended with 5 mM nitrate after day 30, nitrate was reduced with transient formation of nitrite in all four incubations (**Supplementary Figures S2A–D**), indicating that the alkylbenzene-utilizing hNRB demonstrated in **Supplementary Figure S1** were present. When incubations inoculated with concentrated 18PW were amended with 10 mM acetate after day 30, perchlorate was reduced in all four incubations (**Supplementary Figures S2E–H**), indicating that the acetate-utilizing PRB demonstrated in **Figures 1**, **2** were present. Hence, the chemostat cultures contained toluene- and ethylbenzene-oxidizing hNRB, but no PRB capable of using these substrates. PRB oxidizing acetate were present.

### Characterization of VFA-Oxidizing PRB Isolated From MHGC Produced Water

**FIGURE 7:**
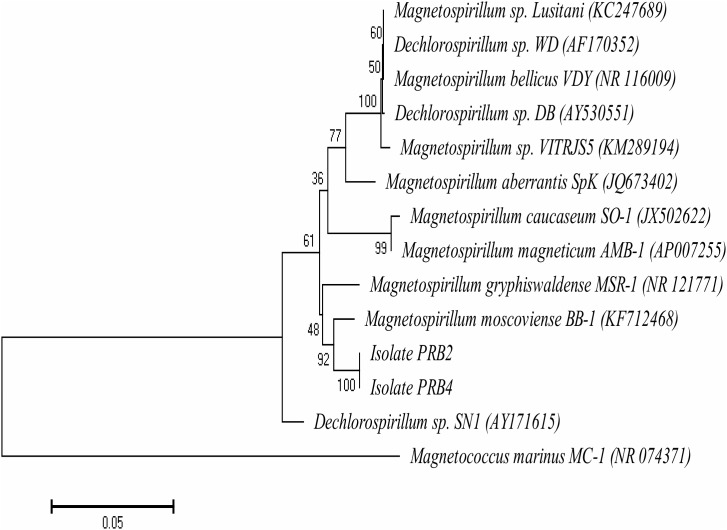
Neighbor-joining phylogenetic tree indicating the affiliation of isolates PRB2 and PRB4 with members of the genus *Magnetospirillum* based on the 16S rRNA gene sequences. Branching points are determined as percentage of bootstrap values based on 1000 replications with those having a cut-off at 50% shown. The scale bar of 0.05 represents the fractional change per nucleotide. The sequence of *Magnetococcus marinus* MC-1 (NR_074371) was used as an outgroup.

Plating of perchlorate-reducing enrichments, as in **Figure 1**, on agar-solidified CSBK medium containing perchlorate and VFA yielded isolates PRB2 and PRB4. 16S rRNA gene sequence analysis indicated that these were closely affiliated with the genus *Magnetospirillum*. A phylogenetic tree of nearly full length 16S rRNA gene sequences (1367 bp) is presented in **Figure 7**. Grouping of the 16S rRNA gene sequences of PRB2 and PRB4 with *Magnetospirillum moscoviense* BB-1 was supported by a bootstrap value of 92%. The 16S rRNA gene sequence similarities of PRB2 and *M. moscoviense* BB-1, and of PRB4 and *M. moscoviense* BB-1 were 93.0 and 92.7%, respectively, whereas the pair-wise similarities to other *Magnetospirillum* strains were between 88.1 and 90.6% (**Supplementary Table S3**). PRB2 and PRB4 shared 99.2% 16S rRNA gene sequence similarity and clustered together with bootstrap support of 100%.

The electron donor and acceptor specificity of PRB2 and PRB4, determined after 7 days of incubation at 30°C, were found to be very similar (**Supplementary Table S4**). Both coupled the reduction of perchlorate to oxidation of acetate, propionate and butyrate, as well as of lactate, succinate, glucose, glutarate and ethanol but not of methanol. Slow growth and perchlorate reduction occurred in medium with a headspace with 20% (v/v) H_2_, whereas no growth or perchlorate reduction occurred with sulfide. Benzene, toluene, or ethylbenzene were not used. Chlorate accumulation was not observed during growth with any of the electron donors utilized. In addition to perchlorate, PRB2 and PRB4 used chlorate, nitrate, and nitrite as electron acceptors with acetate as electron donor. Chlorite likely killed the cells, whereas sulfate and sulfite were not used (**Supplementary Table S4**).

The effect of nitrite or nitrate addition on reduction of perchlorate with lactate as electron donor showed that PRB4 could simultaneously reduce perchlorate and nitrite or nitrate (**Supplementary Figure S3**). When added singly, perchlorate and nitrate were completely reduced at 18 h (**Supplementary Figures S3A,C**), whereas nitrite reduction was complete at 48 h (**Supplementary Figure S3B**). Nitrate addition at 0 or at 6 h did not inhibit perchlorate reduction by PRB4 (**Supplementary Figures S3F,G**). Reduction of perchlorate and nitrite also occurred simultaneously but the time needed for perchlorate reduction (**Supplementary Figures S3D,E**; 48 h) increased by 30 h compared to when only perchlorate was present (**Supplementary Figure S3A**: 18 h).

## Discussion

Much has been learned in the past 20 years on the occurrence and microbial reduction of perchlorate in the environment. Different from nitrate, which is an intermediate in the global microbially catalyzed nitrogen cycle, perchlorate has entered the environment predominantly through human industrial activity, although natural sources of perchlorate may yet be identified (Coates and Achenbach, 2004). Nevertheless, perchlorate-reducing bacteria (PRB) have been found in many environments and have been shown to reduce perchlorate and/or chlorate to chlorite, which is then dismutated to chloride and oxygen through a highly conserved periplasmic chlorite dismutase (Cld). The rapid extracellular metabolism of chlorite is crucial as this reactive and strongly oxidative agent (E^0^’ = +0.78V) is highly biocidal (McDonnell and Russell, 1999). PRB use a variety of organic and inorganic electron donors with acetate being used most frequently (Coates and Achenbach, 2004; Bardiya and Bae, 2011). Use of hydrocarbons, e.g., benzene by *Dechloromonas* strain RCB (Chakraborty et al., 2005) and n-alkanes by *P. chloritidismutans* (Mehboob et al., 2009, 2016), is not wide-spread. Sulfide, when present, is oxidized to inorganic sulfur in preference over the use of organic electron donors like acetate (Gregoire et al., 2014). Microbial communities can protect themselves from the negative effects of perchlorate or chlorate metabolism by harboring the *cld*-gene. Analogs of *cld* are widespread, although it is unlikely that the gene products of all of these do indeed dismutate chlorite (Liebensteiner et al., 2014).

We have successfully enriched and isolated PRB from produced waters of the MHGC field, a shallow, low-temperature heavy oil reservoir, which has been subject to nitrate injection to prevent souring (Voordouw et al., 2009; Agrawal et al., 2012; Shen et al., 2018). Enrichments with perchlorate and VFA were dominated by *Tenericutes*, including the genus *Acholeplasma* (**Supplementary Table S1**). Some of these are also known to reduce nitrate (Sherry et al., 2013; Engelbrektson et al., 2014; Cheng et al., 2016). *Acholeplasma* spp. have been enriched from oilfields (Fida et al., 2016) and marine sediments (Masui et al., 2008; Imachi et al., 2011) and are considered scavengers of dead biomass due to their limited biosynthetic capabilities (Hanajima et al., 2015). Microbial communities present during early stages of perchlorate reduction with VFA were also dominated by *Desulfuromonadales*, as found in other studies where up to a 300-fold increase in this taxon has been reported (Carlström et al., 2016). *Desulfuromonadales* metabolize VFA and can couple the oxidation of VFA to the reduction of sulfur to sulfide, an activity which may occur in enrichments with perchlorate and sulfide (Gregoire et al., 2014). A small fraction of the microbial community enriched with VFA and perchlorate consisted of *Magnetospirillum* spp. (**Supplementary Table S1**: 0.8–0.9%), which was the only taxon obtained by agar plating. Several *Magnetospirillum* spp., including WD, SN1, VITRJS5, and VDY have been shown to reduce perchlorate (Thrash et al., 2010; Jacob et al., 2017). PRB2 and PBR4, isolated here, reduced perchlorate with short chain organic acids, H_2_ and ethanol, as do *Magnetospirillum* spp. WD, SN1, VDY. Other electron donors tested but not utilized for perchlorate reduction were H_2_S, methanol and alkylbenzenes (**Supplementary Table S4**). Meyer-Cifuentes et al. (2017) reported that *Magnetospirillum* sp. 15-1 oxidized toluene with nitrate, but not with perchlorate. Like *M. bellicus* strain VDY (Thrash et al., 2010), PRB2 and PRB4 used chlorate, nitrate, and nitrite as alternate electron acceptors. PRB2 and PRB4 were phylogenetically most similar to *M. moscoviense* BB-1 and metabolically most similar to VDY and WD, although they differentiated themselves from these by reducing nitrate or nitrite concurrently with perchlorate (**Supplementary Figure S3**). Most PRB preferentially reduce nitrate over perchlorate (Chaudhuri et al., 2002; Bardiya and Bae, 2008; Carlström et al., 2013). Sequential reduction of nitrate followed by reduction of perchlorate was also observed in our enrichment cultures (**Figure 2H**) but not in cultures of the isolated *Magnetospirillum* spp. PRB2 and PRB4. This indicates that these were not major components of these cultures, which is in agreement with the microbial community data (**Supplementary Table S1**).

Experiments with batch cultures and bioreactors in which heavy MHGC oil was the only available electron donor indicated absence of PRB capable of coupling the reduction of perchlorate to the oxidation of oil components (**Figures 3**, **5** and **Supplementary Figure S1**). Nitrate was reduced to nitrite and dinitrogen under these conditions, which resulted in removal of alkylbenzenes from the oil (**Supplementary Figure S1**), as found previously (Lambo et al., 2008; Agrawal et al., 2012; Suri et al., 2017). Because perchlorate was not reduced it was inert, i.e., no high-potential electron transport pathways were operating, and sulfide production was observed both in enrichment cultures and bioreactors containing sulfate and perchlorate. Microbial communities in cultures with sulfate and perchlorate were similar to those in cultures with sulfate only (**Figure 4**: clade I), indicating the lack of impact of perchlorate. In contrast, microbial communities in cultures with nitrate were distinct (**Figure 4**: clade II) and were dominated by the hNRB *Thauera* and *Pseudomonas* (**Supplementary Table S2**), which couple reduction of nitrate to nitrite and N_2_ to the oxidation of oil components, especially alkylbenzenes.

The inability of PRB to grow in batch cultures and bioreactors with MHGC oil was due to the absence of usable electron donors, like acetate. Interestingly, acetate has been shown to form in bioreactors with MHGC oil, which were continuously injected with 2 mM sulfate and periodically with 2 mM sulfate and 4 mM nitrate (Callbeck et al., 2013). Transient formation of up to 3 to 4 mM acetate was seen whenever injection of nitrate was stopped. This suggested that upon restoration of sulfate-reducing conditions alkanes were fermented with water to acetate and H_2_ by syntrophic bacteria (Zengler et al., 1999) with the H_2_ being used preferentially for the reduction of sulfate and the acetate accumulating. Acetate was then eventually removed by acetotrophic reactions, as discussed by Callbeck et al. (2013). Use of acetate by acetotrophic SRB is inhibited by light oils and may not occur in light oil-containing oil fields (Menon and Voordouw, 2018), which may also cause acetate accumulation. Indeed, high temperature fields, harboring light oil can have very high concentrations of acetate and propionate in produced waters (Menon and Voordouw, 2018).

Thus, even in the absence of direct hydrocarbon oxidation, there is potential activity of PRB in oil fields. It should be pointed out that injection waters with high VFA concentrations are considered poor fits for treatment with nitrate, because a lot of nitrate would be used oxidizing these aqueous VFA and would thus not make it into the reservoir to prevent souring. In the MHGC field VFA concentrations in produced waters and injection waters are low (of the order of 0.1 mM). Hence most of the nitrate amended into injection water is injected into the reservoir, where it is used for oxidation of oil-alkylbenzenes and sulfide. Because heavy MHGC oil has only low concentrations of alkyl benzenes (of the order of 1 mM), long term injection of nitrate can cause the depletion of alkylbenzenes, which then leads to nitrate breakthrough in producing wells, control of souring and the production of toluene-free oil (Agrawal et al., 2012).

## Conclusion

In conclusion it appears that perchlorate may not be good souring control agent in the MHGC field, where the pros and cons of using nitrate are well understood. Clearly more research is needed to determine whether PRB using oil components as electron donor are present in other fields. A field test in which perchlorate is injected to determine its effectiveness in decreasing souring would also be valuable.

## Author Contributions

GO did the experimental work and wrote a first version of the paper and abstract. GV supervised the work and edited the paper and abstract.

## Conflict of Interest Statement

The authors declare that the research was conducted in the absence of any commercial or financial relationships that could be construed as a potential conflict of interest.
