# Supplementary material for: Comparison of Nitrate and Perchlorate in Controlling Sulfidogenesis in Heavy Oil-Containing Bioreactors
Source: Front Microbiol. 2018 Oct 9;9:2423. doi: 10.3389/fmicb.2018.02423 (PMC6190851; doi:10.3389/fmicb.2018.02423)
Supplement: Supplementary file 1 [file Data_Sheet_1.PDF]

SUPPLEMENTARY INFORMATION TO:

Comparison of nitrate and perchlorate in controlling sulfidogenesis in heavy oil-containing bioreactors

Gloria Ngozi Okpala and Gerrit Voordouw

TABLE S1|Microbial community compositions in batch enrichments with VFA and nitrate (N), perchlorate (P) or perchlorate and nitrate (PN) at different times as in Figure 2. The number of QC reads, some bioinformatic parameters and the fractions (%) of the indicated taxa are shown.

**Sample ID**	**MN_48h**	**MN_336h**	**MP_96h**	**MP_336h**	**MPN_48h**	**MPN_72h**	**MPN_120h**	**MPN_336h**
**Sequence ID**	V50-2784	V50-2785	V50-2778	V50-2781	V50-2770	V50-2771	V50-2772	V50-2773
**# of QC Reads**	14573	22400	14964	11118	19381	16136	33522	14325
**# 0f OTUs**	54	62	78	52	36	36	44	66
**# of taxa**	39	56	69	51	27	30	40	49
**Shannon index**	0.18	1.13	1.93	2.06	0.51	0.49	1.32	1.87
**Taxonomy**	**MN_48h**	**MN_336h**	**MP_96h**	**MP_336h**	**MPN_48h**	**MPN_72h**	**MPN_120h**	**MPN_336h**
Bacteroidia;Paludibacter;	0.01	0.00	12.74	8.92	0.00	0.00	0.13	0.29
Bacteroidia;Petrimonas;	0.01	1.74	0.03	0.00	0.01	0.01	0.11	0.23
Bacteroidia;Proteiniphilum;	0.01	0.25	1.61	1.46	0.02	0.01	0.32	0.40
Bacteroidia;Rikenellaceae;vadinBC27	0.02	0.39	0.24	0.16	0.02	0.04	0.12	11.56
Sphingobacteriia;WCHB1-69;	0.08	2.57	7.40	14.28	0.22	0.27	4.03	13.81
Clostridia;Christensenellaceae;	0.00	0.04	0.01	0.08	0.02	0.13	0.38	2.00
Clostridia;Dethiosulfatibacter;	0.00	0.06	0.00	0.01	0.00	0.00	0.13	0.74
Clostridia;Tissierella;	0.10	0.19	0.03	0.00	0.07	0.05	0.26	0.20
Clostridia;Acidaminobacter;	0.00	0.00	7.91	4.10	0.00	0.00	0.00	0.00
Clostridia;Fusibacter;	0.01	0.01	0.24	0.23	0.01	0.06	0.85	0.46
Clostridia;Anaerovorax;	0.00	0.71	0.16	1.92	0.02	0.13	1.55	0.71
Clostridia;Syntrophomonas;	0.06	3.72	0.01	0.00	0.00	0.00	0.00	0.00
Alphaproteobacteria;Magnetospirillum;	0.00	0.00	0.84	0.91	0.03	0.04	0.28	0.02
Betaproteobacteria;Thauera;	2.06	10.53	1.12	0.59	16.04	11.76	21.22	0.72
Deltaproteobacteria;Desulfuromonadales;	0.01	0.00	39.21	2.21	0.00	0.00	0.00	0.00
Gammaproteobacteria;Pseudomonas;	96.99	73.82	0.14	0.11	83.15	86.44	58.53	2.18
Spirochaetes;LNR_A2-18;	0.00	0.00	0.01	0.34	0.00	0.00	0.00	1.53
Spirochaetes;Spirochaeta;	0.19	3.16	2.82	5.24	0.12	0.34	1.22	16.32
Tenericutes;Acholeplasma;	0.15	1.70	4.97	23.05	0.16	0.56	10.42	47.23
Tenericutes;Mollicutes;EUB33-2;	0.00	0.28	19.26	34.00	0.00	0.00	0.00	0.00

TABLE S2 | Microbial community compositions of batch incubations in the presence of oil (O), sulfate (S), nitrate (N) and perchlorate (P), as indicated. The numbers of QC reads, some bioinformatic parameters and the fractions (%) of the indicated taxa are shown.

#		PSNO T=9d	SNO T=9d	SNO T=123d	PSNO T=123d	PSNO T=65d	PSNO T=85d	SNO T=65d	SNO T=85d	PO T=123d	PSO T=123d	PSO T=85d	SO T=123d
	**Sequence ID**	V64_4109	V64_4112	V66_4251	V66_4253	V64_4110	V64_4111	V64_4113	V64_4114	V66_4254	V66_4252	V64_4115	V66_4250
	**# of QC Reads**	57231	66669	28254	31951	29220	49339	43903	56034	29865	65303	43493	27962
	**# OTUs**	115	124	215	148	156	175	160	163	287	322	325	311
	**# Taxa**	97	96	151	127	129	136	136	136	269	279	279	286
	Shannon index	0.92	0.76	1.91	2.04	1.76	1.90	1.98	2.00	3.61	3.58	2.82	3.93
	#Taxonomy												
1	Bacteria;Proteobacteria;Betaproteobacteria;Rhodocyclales;Rhodocyclaceae;Thauera;	54.03	81.58	54.78	44.09	52.00	46.21	45.05	47.58	7.40	0.63	2.69	0.07
2	Bacteroidete;Sphingobacteriia;Sphingobacteriales;NAWCHB1-69;	0.54	1.71	21.56	15.37	28.58	30.72	30.65	28.17	11.21	5.91	7.08	14.18
3	Bacteria;Firmicutes;Clostridia;Clostridiales;Peptococcaceae;Pelotomaculum;	0.00	0.00	0.12	19.92	0.01	0.00	0.00	0.00	0.00	24.31	39.96	10.97
4	Bacteria;Proteobacteria;Gammaproteobacteria;Pseudomonadaceae;Pseudomonas;	42.55	12.14	1.89	1.43	0.69	1.23	1.46	2.13	6.41	3.12	0.60	4.50
5	Bacteria;Proteobacteria;Betaproteobacteria;Burkholderiales;Alcaligenaceae;Bordetella;	0.98	1.90	0.63	0.67	1.85	1.36	3.08	1.83	9.88	6.01	0.00	5.00
6	Bacteria;noCandidate-division-WS6;	0.00	0.00	1.94	1.93	6.12	6.74	3.82	3.21	0.15	3.02	0.98	1.62
7	Bacteria;Chloroflexi;Anaerolineae;Anaerolineales;Anaerolineaceae;	0.02	0.04	5.35	2.08	1.72	1.76	1.54	1.52	2.99	3.64	1.40	3.69
8	Bacteria;Chlorobi;Ignavibacteria;Ignavibacteriales;NAIheB3-7;	0.00	0.01	4.61	3.86	2.46	4.09	2.24	2.60	0.08	0.03	0.01	0.08
9	Bacteria;Proteobacteria;Deltaproteobacteria;Syntrophaceae;Smithella	0.00	0.00	0.00	0.00	0.00	0.00	0.00	0.00	6.46	4.48	5.76	2.76
10	Bacteria;Bacteroidetes;Sphingobacteriia;Sphingobacteriales;NAB01R012;	0.00	0.00	0.00	0.00	0.00	0.00	0.00	0.00	6.73	6.39	0.49	1.34
11	Bacteria;Thermotogae;Thermotogae;Thermotogales;Thermotogaceae;Mesotoga;	0.01	0.02	0.02	0.01	0.01	0.01	0.01	0.02	1.39	3.54	2.19	3.36
12	Bacteria;Tenericutes;Mollicutes;Acholeplasmatales;Acholeplasmataceae;Acholeplasma;	0.17	0.42	0.12	0.03	0.00	0.00	0.14	0.31	2.62	1.22	1.84	3.30
13	Bacteria;Microgenomates;	0.00	0.00	0.71	2.85	1.08	1.88	0.43	0.56	0.14	1.28	0.18	0.37
14	Archaea;NAWoesearchaeota-(DHVEG-6);	0.00	0.00	0.00	0.00	0.00	0.00	0.00	0.00	3.44	1.21	4.37	0.22
15	Bacteria;Firmicutes;Clostridia;Clostridiales;Syntrophomonadaceae;Syntrophomonas;	0.00	0.00	0.00	0.00	0.00	0.00	0.00	0.00	6.85	0.15	0.03	0.00
16	Bacteria;Proteobacteria;Deltaproteobacteria;Desulfarculaceae;Desulfarculus;	0.00	0.00	0.00	0.00	0.00	0.00	0.00	0.00	0.00	3.08	2.58	1.05
17	Bacteria;Proteobacteria;Gammaproteobacteria;Enterobacteriaceae;Raoultella;	0.00	0.00	0.27	0.14	0.00	0.00	0.00	0.00	2.90	1.36	0.00	1.40
18	Bacteria;Proteobacteria;Deltaproteobacteria;;Desulfomicrobiaceae;Desulfomicrobium;	0.00	0.00	0.02	0.00	0.00	0.00	0.00	0.00	0.88	1.56	1.00	2.43
19	Bacteria;Firmicutes;Clostridia;Clostridiales;NAFamily-XIII;Anaerovorax;	0.00	0.00	0.00	0.00	0.00	0.00	0.00	0.00	0.61	1.78	1.38	1.71
20	Bacteria;Proteobacteria;Deltaproteobacteria;Desulfovibrionaceae;Desulfocurvus;	0.07	0.18	0.01	0.01	0.16	0.11	0.45	0.21	0.07	0.27	1.23	0.81
21	Bacteria;Firmicutes;Bacilli;Lactobacillales;Streptococcaceae;Streptococcus;	0.00	0.00	0.01	0.00	0.03	0.01	0.01	0.00	2.68	0.07	0.00	0.03
22	Bacteria;Proteobacteria;Deltaproteobacteria;Desulfobacterales;Desulfobacteraceae;	0.00	0.00	0.00	0.00	0.00	0.00	0.00	0.00	0.00	0.45	0.46	1.19

Table S3 | 16S rRNA gene similarity and sequence distance among isolates PRB2, PRB4 and *Magnetosprillum* strains.

	**16S rRNA gene sequence similarity (%)**												
**Strains**	SpK (JQ673402)	VDY (NR_116009)	SO-1 (JX502622)	MSR-1 (NR_121771)	AMB-1 (AP007255)	BB-1 (KF712468)	Isolate PRB2	Isolate PRB4	VITRJS5 (KM289194)	*M. lusitani* (KC247689)	WD (AF170352)	DB (AY530551)	SN1 (AY171615)
M. aberrantis SpK (JQ673402)	ID	94.9	93.8	96.0	95.6	93.6	88.4	88.1	61.6	88.9	96.4	96.8	94.3
M. bellicus VDY (NR_116009)	0.029	ID	94.1	94.5	93.9	95.1	90.7	90.4	63.9	92.8	98.0	98.0	94.1
M. caucaseum SO-1 (JX502622)	0.042	0.046	ID	93.8	97.2	95.7	90.2	89.9	61.1	89.6	93.9	93.8	92.5
M. gryphiswaldense MSR-1 (NR_121771)	0.034	0.038	0.051	ID	95.8	95.3	90.6	90.3	60.6	88.6	95.9	96.4	95.7
M. magneticum AMB-1 (AP007255)	0.041	0.043	0.003	0.047	ID	93.7	88.6	88.3	60.1	87.9	95.7	95.7	94.5
M. moscoviense BB-1 (KF712468)	0.044	0.033	0.055	0.026	0.054	ID	93.0	92.7	62.3	90.5	94.9	95.0	94.5
Isolate PRB2	0.048	0.034	0.055	0.028	0.052	0.017	ID	99.2	65.1	94.2	90.1	89.9	89.7
Isolate PRB4	0.048	0.034	0.055	0.028	0.052	0.017	0.000	ID	64.9	94.0	89.7	89.6	89.3
Magnetospirillum sp. VITRJS5 (KM289194)	0.029	0.004	0.051	0.042	0.047	0.038	0.039	0.039	ID	68.4	63.3	63.3	60.5
Magnetospirillum sp. *lusitani* (KC247689)	0.029	0.000	0.046	0.038	0.043	0.033	0.034	0.034	0.004	ID	91.9	91.9	88.1
Dechlorospirillum sp. WD (AF170352)	0.029	0.000	0.046	0.038	0.043	0.033	0.034	0.034	0.004	0.000	ID	99.4	95.7
Dechlorospirillum sp. DB (AY530551)	0.028	0.001	0.047	0.037	0.044	0.032	0.036	0.036	0.005	0.001	0.001	ID	96.0
Dechlorospirillum sp. SN1 (AY171615)	0.051	0.042	0.061	0.043	0.058	0.032	0.036	0.036	0.046	0.042	0.042	0.041	ID
	**16S rRNA gene sequence distance**												

TABLE S4 | Anaerobic growth tests of perchlorate-reducing *Magnetospirillum* spp. PRB2 and PRB4 with different electron donors and electron acceptors. The numbers indicate growth as the maximum protein content in µg/mL. Alternatively, growth (+) or no growth (-) was evaluated qualitatively.

**Electron Donor***	**PRB2**	**PRB4**
Acetate	453	425
Propionate	697	744
Butyrate	799	645
Lactate	613	683
Glutarate	120	52
Succinate	742	592
Glucose	159	30
Ethanol	699	733
Methanol	-	-
H_2	+	+
Sulfide	-	-
Benzene	-	-
Toluene	-	-
Ethylbenzene	-	-
m-Xylene	-	-
**Electron acceptor****		
Perchlorate	+	+
Chlorate	+	+
Chlorite	-	-
Nitrate	+	+
Nitrite	+	+
Sulfate	-	-
Sulfite	-	-

*Perchlorate was the electron acceptor
**Acetate was the electron donor

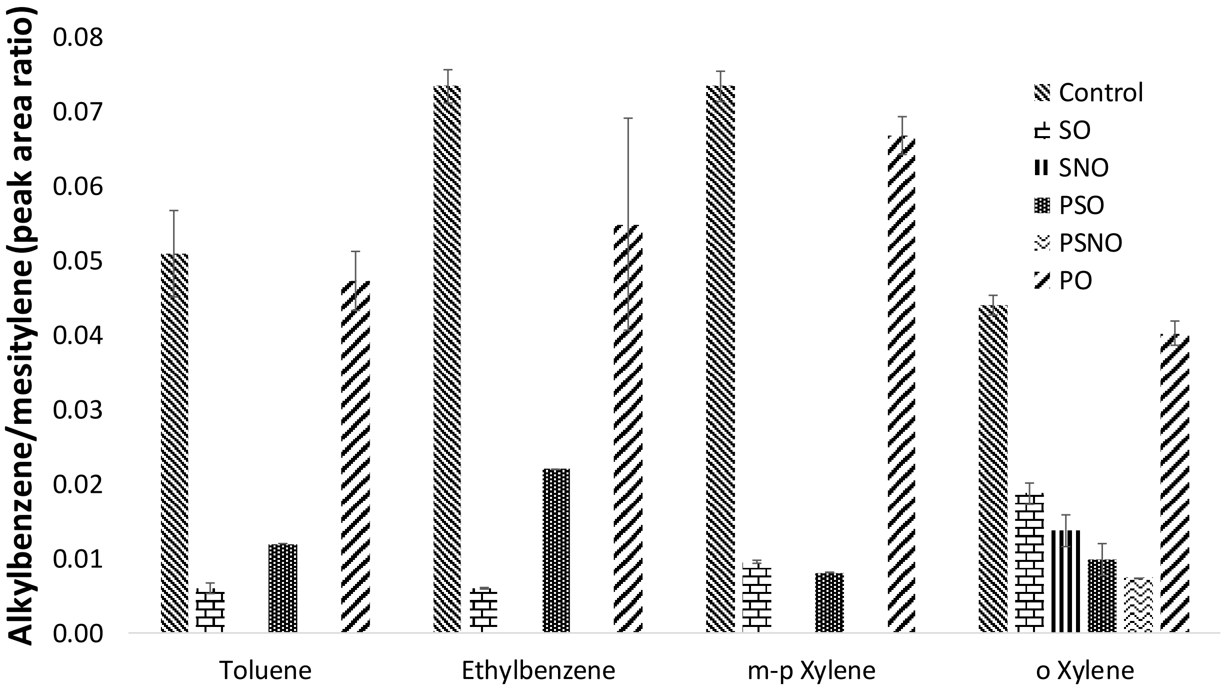

FIGURE S1│Gas chromatography-mass spectrometry analysis of the alkylbenzenes in MHGC oil in incubations of Figure 3 remaining after 123 days. The fraction of alkylbenzenes remaining was calculated as the ratio of the peak area of each alkylbenzene to that of the added internal standard mesitylene (1,3,5-trimethylbenzene). Data presented are the mean and average deviation for duplicate measurements.

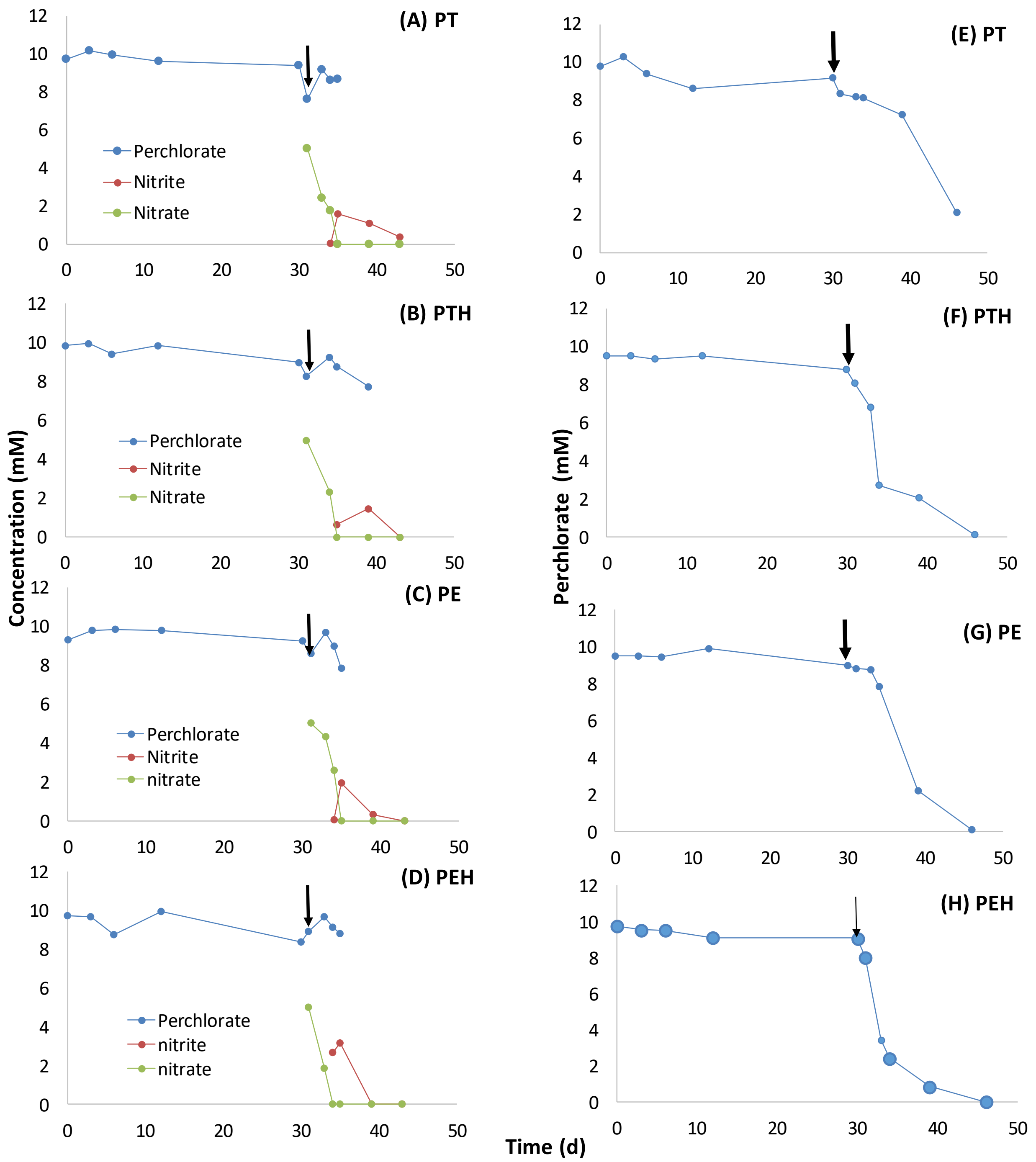

FIGURE S2| Attempts at enrichment of alkylbenzene-oxidizing PRB. The alkylbenzenes toluene (T) and ethylbenzene (E) were added directly to 50 mL CSBK or to 1 mL HMN (H) in 50 mL CSBK medium with 10 mM perchlorate (P) and inoculated with 5 mL of a nitrate-reducing chemostat culture growing on toluene or ethylbenzene (A-D) or with 1 mL of 20-fold concentrated 18PW (E-H). The arrows (↓) indicate the point where nitrate (A-D) or acetate (E-H) was injected into the serum bottles. The concentrations of perchlorate, nitrate and nitrite are indicated in (A-D) and of perchlorate in (E-H). Data presented are averages of duplicate incubations

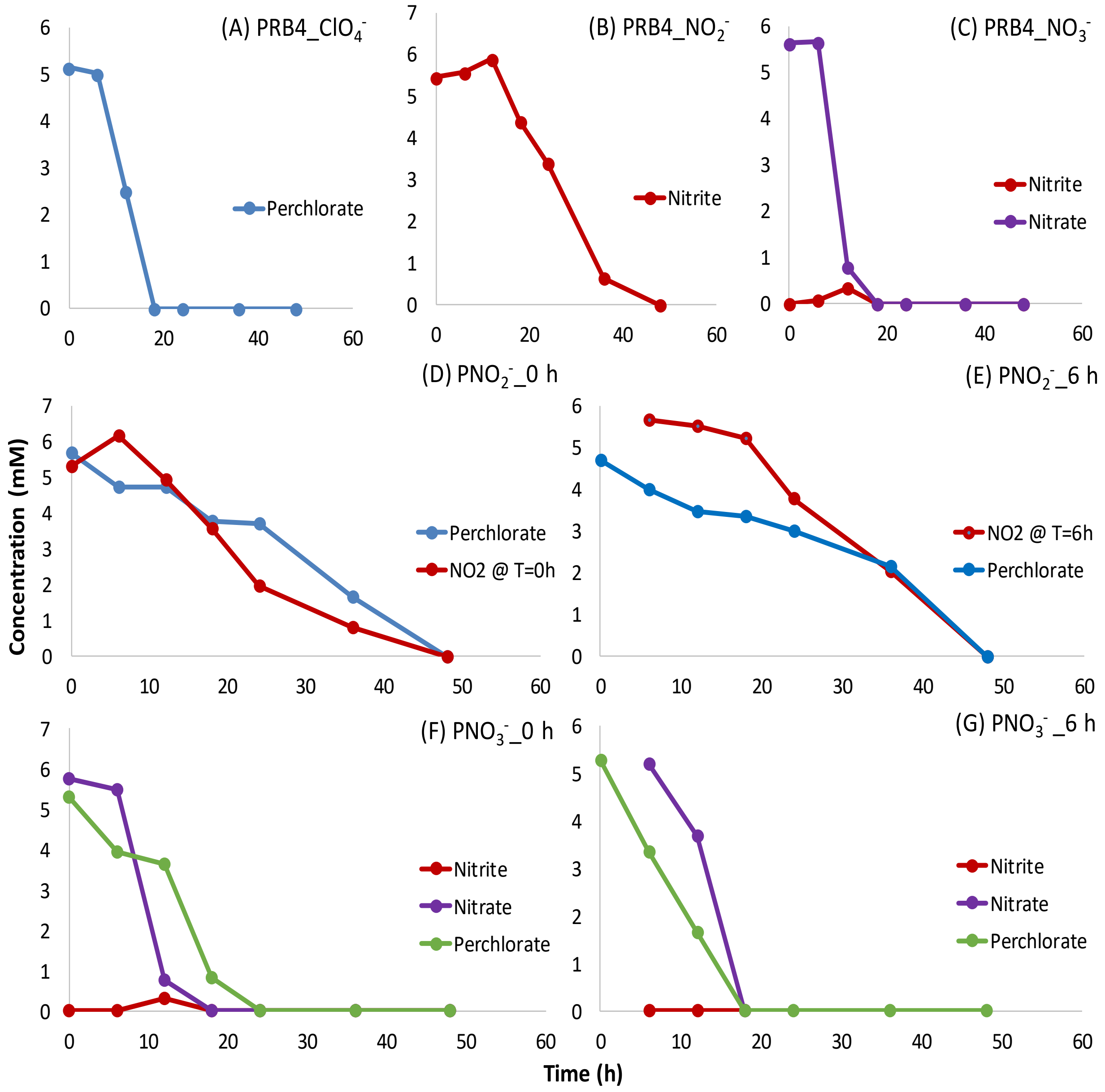

FIGURE S3 | Effect of nitrate or nitrite on perchlorate reduction by isolate PRB4. Growth of PRB4 with perchlorate (A), nitrite (B), or nitrate (C). Perchlorate reduction by PRB4 following the addition of nitrite at time T = 0 h (D) or 6 h (E) and nitrate at T = 0 h (F) or 6 h (G). Electron donor used was 20 mM lactate.
